# Literary Intelligence

**Published:** 1893-01-14

**Authors:** 


### LITERARY INTELLIGENCE.

The third and fourth volumes, with portfolio of plans (completing the work), of Mr. Henry C. Burdett's "Hospitals and Asylums of the World," will be published by Messrs. J. and A. Churchill and the Scientific Press, about the end of this month. Vol. III. consists of hospitals, and deals with their history and administration in all countries throughout the world. Vol. IV. relates to hospital construction, and contains exhaustive bibliography and plans. The plans in the portfolio comprise those of the principal British, Colonial, American, and foreign hospitals, convalescent institutions, nurses' home, and medical school buildings, accurately drawn to the same scale, and replete with the fullest and most detailed information. It also contains plans of every hospital in London in the jubilee year of her Majesty's reign.

